# Impact of burnout on depression among nurses at a private hospital in Johannesburg, South Africa

**DOI:** 10.4102/safp.v66i1.5906

**Published:** 2024-08-28

**Authors:** Ongeziwe Dyasi, Emmanuel E.-O. Agbenyeku, Anesu G. Kuhudzai, Teboho A. Moloi

**Affiliations:** 1 Department of Environmental Health, Faculty of Health Sciences, University of Johannesburg, Johannesburg, South Africa; 2 Statistical Consultation Services, University of Johannesburg, Johannesburg, South Africa; 3 Postgraduate School, University of Johannesburg, Johannesburg, South Africa

**Keywords:** burnout, depersonalisation, emotional exhaustion, factors, nursing practitioners, private hospital, reduced personal accomplishment, South Africa

## Abstract

**Background:**

Burnout is a syndrome that is understood as emanating from chronic workplace stressors that have not been managed successfully. Little is known about the causes of burnout among nurses in South Africa. The study aimed to determine the prevalence of burnout and its impact on depression and assess the relationship between burnout and depression among nurses at a Johannesburg private hospital.

**Methods:**

Nurses at a private hospital in Johannesburg were asked about their exposure to depression and burnout using a closed-ended questionnaire as part of a quantitative, cross-sectional study design. A *p*-value < 0.05 was considered statistically significant. The respondents were selected using the simple-random sampling method. The collected data were analysed using IBM-SPSS version 28.

**Results:**

The study involved 112 nurses, of whom 95 (84.8%) were females. Most of the nurses, that is, 56 (50.0%) were registered nurses. Emotional exhaustion (*p* = 0.001) and depersonalisation (*p* = 0.001) were significantly associated with depression. Work experience (*p* = 0.001) and depersonalisation (*p* = 0.002) had an impact on depression.

**Conclusion:**

The study revealed a high prevalence of burnout among nurses at a Johannesburg private hospital. The study found that depression was significantly associated with emotional exhaustion and depersonalisation. The study also found that work experience and depersonalisation have an impact on depression.

**Contribution:**

The study’s recommendations can help mitigate burnout and improve the well-being of nurses, ultimately enhancing the quality of healthcare services provided at the hospital.

## Introduction

Nurses spend a great deal of time with patients and, as a result, are continuously exposed to various strains and stressors of attending to sick and dying patients. If left unchecked, these daily stressors can cause burnout.^1^ Burnout is a prevalent psychosocial experience that nurses and other health personnel go through.^2^ Burnout has also been defined as ‘a syndrome conceptualised as resulting from chronic workplace stress that has not been successfully managed’.^3^ There are three dimensions encompassing burnout: (1) emotional exhaustion (EE), which is characterised by a feeling of mental and physical overexertion accompanied by a lack of energy; (2) depersonalisation (DP), which is characterised by negative feelings and attitudes towards patients and colleagues alike, leading to emotional detachment; and (3) reduced personal accomplishment (PA), which is the extent a person perceives himself or herself as performing poorly on worthwhile tasks.^4^ The pooled prevalence of burnout symptoms was 11.23% overall amongst nurses worldwide.^5^ There were notable distinctions observed throughout specialisations, geographical areas, and burnout measuring types. The prevalence of burnout symptoms was highest in sub-Saharan Africa and lowest in Europe and Central Asia. Burnout affects employees negatively because it causes numerous physical and mental problems on individuals, thereby decreasing organisational productivity and the quality of care rendered to patients.^6^ Burnout and depression have a negative impact on each other, according to Koutsimani et al.^7^

Depression is one of the conditions that nurses are susceptible to, which according to Tran et al.,^8^ is the most common and most cited in psychological research involving healthcare providers. According to World Health Organization, depression can either last long or be recurrent.^9^ Still, in either case, it will likely substantially impair an individual’s functioning in the workplace and in daily life.

Research conducted in South Africa on the relationship between personal and work-related stress, job satisfaction, burnout and the general health of hospital nurses has contributed notable insights. For instance, a study by Tran et al. sought to determine whether personal stress could predict burnout, job satisfaction and general well-being better than work-related stress.^8^ The study found that personal and work-related stress was a result of the difficulty to balance work with family responsibilities, and this difficulty had several negative outcomes, including depression.^8^

Meanwhile, information on the precise causes of burnout among nurses in South Africa is still insufficient.^10^ What is indisputable, though, is that nurses are predisposed to burnout because of the very nature of their profession, which is generally understood as stressful and demanding both physically and emotionally.^11^ In South Africa, the demanding roles of nurses are exacerbated by the increasing population resulting in disproportionate workload, long working hours, attending to high-risk cases daily, the patients’ varied emotional care needs, and numerous other factors inherent in nursing work.^12^

This study hopes to contribute to the existing efforts of building a healthy workforce that is capable of providing a high-quality nursing service in South Africa by investigating the prevalence of burnout and the associated contributions to burnouts among nurses at a Johannesburg private hospital.

## Research methods and design

### Research design

The authors used a cross-sectional, quantitative design for their investigation. The cross-sectional study design is one type of the observational study design. In a cross-sectional study, the researcher simultaneously assesses the study participants’ exposures and outcomes.^13^

### Study setting

The research was conducted at a private hospital in Midrand, Johannesburg, South Africa. This hospital is in a popular area for business and development. Because one of the researchers or authors works at the hospital, it was expected that respondents would be easily accessible on this study location. The hospital that was chosen has 200 beds and is multidisciplinary.^14^

### Study population

The population of the study was 300 full-time nurses, both males and females, who worked at a private hospital in Johannesburg. In all, 112 respondents participated in the study. Nursing students and nursing managers were excluded from the study because they might not have revealed a true picture of what was being researched.

### Sample size calculation

The sample size was calculated using a Statistics Kingdom sample size online calculator (statskingdom.com/sample_size_regression.html). The sample size of 112 respondents was decided using 80% study power, 95% confidence interval, medium effect size of 0.39 and 9 predictors.

### Sampling procedure

Respondents were selected by the simple random sampling technique, as each nurse had an equal chance of being selected. The questionnaire was distributed through an online survey as well as distributing physical copies in order to attain the required sample size. The online survey was shared by email to all nurses in management because they had access to laptops or desktops, and all the nurses who were in non-management position completed the paper-based questionnaire. A questionnaire was used as instrument of choice because it ensures anonymity and confidentiality. To also ensure the anonymity and confidentiality, questions such as names of respondents were not asked.

### Data collection

The questionnaire consisted of three sections. Section A included questions on the biographical details assessing six items such as gender, qualifications of the nurse, age, working experience, working hours per shift, and working days per week. Section B assessed burnout by using the Maslach Burnout Inventory with three dimensions, namely EE, DP, and PA.^15^ Items responses were recorded in a scale anchored by 0 = ‘never’ and 6 = ‘every day’. Section C measured depression using the Beck Depression Inventory (BDI) instrument.^16^ The timeframe for recruiting and gathering data was October 2021 – August 2022. The consent form was signed by each respondent prior to them answering the questionnaire. Completing the questionnaire took 15 min or less. The questionnaire was administered in English, and to guarantee that there was no communication gap, a selection criterion question was included to make sure all respondents could read English. Thirty participants were given physical questionnaires as part of a pilot study and these respondents were not considered as part of the research.

### Data analysis

Data were captured and analysed using IBM^®^ SPSS^®^ statistics version 28. The descriptive statistics such as frequencies and percentages were calculated for nominal and ordinal variables, while means and standard deviations (s.d.) were used to describe the numerical data. The prevalence of burnout as measured by ‘emotional exhaustion, depersonalisation and reduced personal accomplishment’ and depression among nurses at a Johannesburg private hospital were determined using custom tables, means and s.d. The relationship between burnout and depression among nurses working at a private hospital in Johannesburg was evaluated using correlation analysis. The impact of burnout on depression among nurses working at a private hospital in Johannesburg was determined using multiple linear regression analysis.

### Informed consent and data privacy

Prior to answering the questionnaire, respondents were required to thoroughly read and answer the consent form. Respondents in the survey were assured that the data would be solely for research purposes, treated as anonymous and confidential. The data privacy was ensured by keeping the data in a password-protected laptop, and the password was only known to researchers.

### Ethical considerations

Ethical clearance to conduct this study was obtained from the Faculty of Health Sciences Research Ethics Committee (NHREC Registration: REC241112-035). The respondents had to complete a consent form to participate in the study.

## Results

This section presents the empirical results of the study. These results are presented in the form of tables and figures. The data analysis includes frequencies, custom tables, reliability analysis, correlation analysis and multiple linear regression analysis.

Table 1 presents the frequency analysis of the demographic information of the study. The variables included are gender, qualification, age, years of service, hours worked per shift and number of days worked per week.

**TABLE 1:** Biographical information.

Biographical data	Category	*n*	%	Mean	Min	Max
Gender	Male	17	15.2	-	-	-
	Female	95	84.8	-	-	-
	Total	112	100	-	-	-
Qualification (rank)	Registered nurse	56	50.0	-	-	-
	Enrolled nurse	33	29.5	-	-	-
	Nursing assistant	23	20.5	-	-	-
Age	-	-	-	39.42	23	63
Years of service	-	-	-	13.63	1	40
Hours worked per shift	-	-	-	11.75	3	12
Days worked per week	-	-	-	5.57	2	7

Min, minimum; Max, maximum.

The data displayed in Table 1 show that 112 nurses participated in the survey. A large majority of this study’s respondents (84.8%, *n* = 95) were female and only 15.2% (*n* = 17) were male. About half of the nurses (50.0%, *n* = 56) were registered nurses, followed by 29.5% (*n* = 33) who were enrolled nurses, and 20.5% (*n* = 23) were nursing assistants.

The age distribution of the nurses fell between 23 years and 63 years with a mean age of 39.42 years. The years of service corresponded with the age distribution, in that there was a 40-year gap in both instances. Years of service ranged between 1 year and 40 years, with an average work experience of 14 years (M = 13.63). On average, the nurses worked a 12-h shift (M = 11.75) and about 6 days per week (M = 5.57).

Table 2 reveals the prevalence of depression and burnout as measured by EE, DP and reduced PA.

**TABLE 2:** Level of emotional exhaustion, depersonalisation, reduced personal accomplishment and depression.

Mental health status	Category	*n*	%
Level of emotional exhaustion	Low-level burnout (17 or less)	26	23.2
	Moderate-level burnout (18 and 29)	26	23.2
	High-level burnout (30 and greater)	60	53.6
	Total	112	100
Level of depersonalisation	Low-level burnout (5 or less)	11	9.8
	Moderate burnout (6 and 11)	14	12.5
	High-level burnout (12 and greater)	87	77.7
	Total	112	100
Level of reduced personal accomplishment	Low-level burnout (40 and greater)	16	14.3
	Moderate burnout (34–39)	15	13.4
	High-level burnout (33 or less)	81	72.3
	Total	112	100
Level of depression	Minimal depression (0–13)	51	45.5
	Mild depression (14–19)	16	14.3
	Moderate depression (20–28)	25	22.3
	Severe depression (29–63)	20	17.9
	Total	112	100

Results emanating from Table 2 suggest that the majority of respondents suffer from high-level of burnout through EE (53.6%, *n* = 60), 23.2% (*n* = 26) implies moderate burnout, while an equal proportion (23.2%, *n* = 26) reveals low levels of burnout. These results suggest that all the surveyed nurses may have experienced some level of burnout in their job.

Results from the ‘depersonalisation’ composite variable as presented in Table 2 confirm the EE results. However, high-level burnout seemed more prevalent for the DP composite variable (77.7%, *n* = 87) compared to EE composite variable (53.6%, *n* = 60). Again, these results confirm that all the respondents may have experienced some level of burnout in their job.

Results emanating from this composite variable (reduced PA), displayed in Table 2, suggest there could be a high prevalence of high-level burnout (72.3%, *n* = 81) among the respondents. Moderate burnout is likely experienced by 13.4% (*n* = 15), while 14.3% (*n* = 16) of the respondents likely experienced low-level burnout. These results also suggest that all the respondents may have experienced burnout to a particular degree, although most possibly experienced high-level burnout.

The findings from the BDI revealed that certain characteristics, attitudes, and symptoms that may be attributed to depression range from minimal to severe. A notable number (17.9%, *n* = 20) of the respondents may be deemed as showing characteristics of severe depression. The results also indicate that 22.3%(*n* = 25) showed characteristics that might signal moderate depression. On the other hand, a significant proportion (45.5%, *n* = 51) showed characteristics that could suggest minimal depression.

### Reliability results

In this study, Cronbach’s alpha was employed to estimate the reliability of the measurement scales, namely DP, reduced PA, EE and depression. Cronbach’s alpha reliability coefficient is measured between 0 and 1. The Cronbach’s alpha coefficient value above 0.7 is considered highly reliable and acceptable.^17^

It can be seen from Table 3 that Cronbach’s alpha value of each construct exceeded the required level of 0.70, supporting the internal consistency and high reliability of the study constructs.

**TABLE 3:** Reliability of the measurement scales.

Construct	Cronbach’s alpha coefficient	Number of items
Emotional exhaustion	0.909	7
Depersonalisation	0.838	7
Reduced PA	0.847	8
Depression	0.911	21

PA, personal accomplishment.

### Correlation analysis

To assess the relationship between burnout as measured by ‘emotional exhaustion, DP and reduced personal accomplishment’^15^ and depression among nurses working at a private hospital in Johannesburg, correlation analysis was performed; readers should observe that in this case burnout is a second order construct measured through EE, DP and reduced PA. Correlation analysis is measured by the correlation coefficient (rho).^18^

Table 4 shows that there is a statistically significant (*p* < 0.05) strong positive correlation between EE and depression (*r* = 0.582). Additionally, there is a strong positive correlation between DP and depression (*r* = 0.608).

**TABLE 4:** Correlation between emotional exhaustion, depersonalisation, reduced personal accomplishment and depression.

Correlations	Emotional exhaustion	Depersonalisation	Reduced personal accomplishment	Depression
**Emotional exhaustion**				
Pearson correlation	1	-	-	-
Sig. (2-tailed)	-	-	-	-
*N*	112	-	-	-
**Depersonalisation**		-	-	-
Pearson correlation	0.800**	1	-	-
Sig. (2-tailed)	< 0.001	-	-	-
*N*	112	112	-	-
**Reduced personal accomplishment**				
Pearson correlation	−0.099	−0.192*	1	-
Sig. (2-tailed)	0.300	0.043	-	-
*N*	112	112	112	-
**Depression**				
Pearson correlation	0.582**	0.608**	−0.141	1
Sig. (2-tailed)	< 0.001	< 0.001	0.137	-
*N*	112	112	112	112

Sig., significance.

* , Correlation is significant at the 0.05 level (2-tailed).

** , Correlation is significant at the 0.01 level (2-tailed).

### Multiple linear regression analysis

Multiple linear regression analysis was conducted in this study to explore the impact of burnout on depression among nurses working at a private hospital in Johannesburg. Multiple linear regression analysis is a statistical technique used to analyse linear relationships between a dependent variable and two or multiple independent variables.^19^

The model meant to determine the impact of burnout on depression among nurses working at a private hospital in Johannesburg was statistically significant (*p* < 0.05).

For appropriate conclusion(s) to be drawn from the multiple linear regression analysis output, assumptions of multicollinearity, normality and homoscedasticity of the residuals need to be examined and met. These assumptions apply to the dependent and independent variables and the relationship as a whole.^20^

Multicollinearity happens when independent variables in the regression model are highly correlated to each other. Multicollinearity is evaluated by tolerance values of less than 0.10 and variance inflation factor (VIF) values of greater than 10.^19^

It is evident from Table 5 that all tolerance values are above 0.10 and the VIF values are below 10 suggesting that the model does not suffer from multicollinearity.

**TABLE 5:** Coefficients table.

Model	Unstandardised coefficients		Standardised coefficients	*t*	Sig.	95% CI for B		Collinearity statistics	
	B	Standard error	Beta			Lower bound	Upper bound	Tolerance	VIF
**Coefficients†**									
(Constant)	0.450	3.312	-	0.136	0.892	−6.120	7.020	-	-
Emotional exhaustion	0.123	0.121	0.119	1.017	0.311	−0.117	0.363	0.344	2.910
Depersonalisation	0.439	0.135	0.374	3.255	0.002	0.171	0.706	0.354	2.825
Reduced personal accomplishment	−0.028	0.078	−0.026	−0.362	0.718	−0.182	0.126	0.939	1.064
Working experience	0.468	0.088	0.397	5.321	< 0.001	0.294	0.643	0.843	1.187

Sig., significant; CI, confidence interval; VIF, variance inflation factor.

† , Dependent variable: Depression.

**FIGURE 1:**
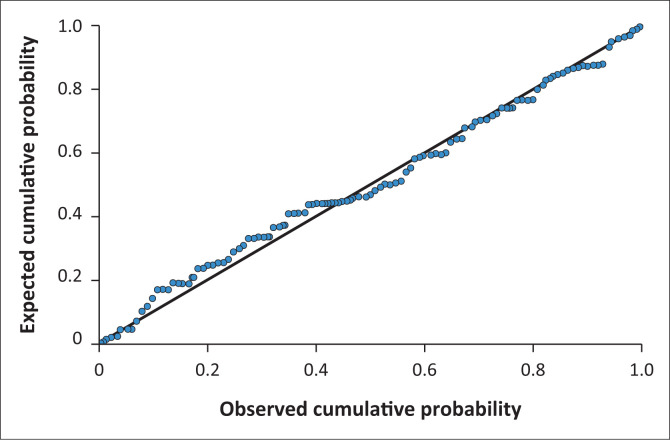
Normal P-P Plot of regression standardised residual – Dependent variable: Depression.

As shown in Figure 1, the normal P-P plot for the model suggested that the assumption of normality of the residuals has not been violated.

**FIGURE 2:**
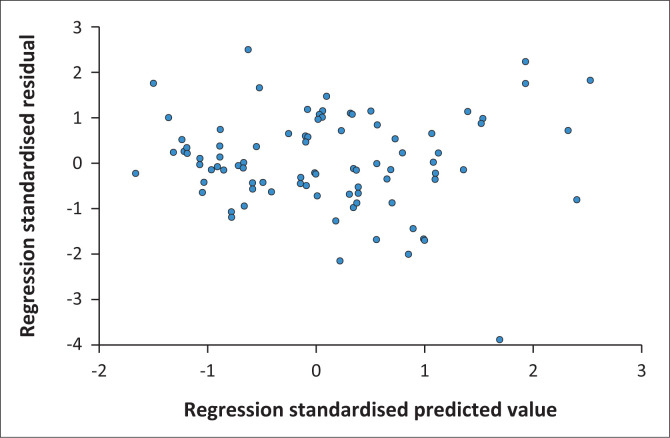
Scatterplot – Dependent variable: Depression.

The scatterplot presented in Figure 2 shows that the homoscedasticity assumption had been met because there is no pattern to the data distribution and residuals are randomly around the horizontal line through zero of the residual’s plots.

Now that the multiple linear regression model has now adhered to all the assumptions^22^, the beta coefficients can now be interpreted.

Evaluation of the independent variables presented in Table 5 suggests that DP (*p* = 0.002) and working experience (*p* < 0.05) had a statistically significant impact on depression.

When the multiple linear regression model is conducted, an *R*^2^ (Coefficient of Determination) is presented to assess how well the model explains the dependent variable.

The coefficient of determination was 0.523, indicating that 52.3% of the variability in depression (dependent variable) has been significantly explained by the independent variables (DP and working experience).

## Discussion

The descriptive statistics included gender, age, qualification or rank, years of service, hours worked per shift, and days worked per week. A large majority of the respondents were women (84.8%), which is hardly surprising because nursing remains a female-dominated profession in South Africa.^21^ In terms of the rank or qualification, around half of the nurses were registered nurses and the rest were either enrolled nurses or nursing assistants.

In this study, the ages of the nurses fell between 23 years and 63 years with the mean age of 39.42 years. This means most nurses still had many years of service ahead, before they could reach the age of retirement. The average work experience of the nurses was 14 years. When the mean age and average work experience are considered together, most nurses can be described as being in the mid-career stage. The nurses worked mostly 12-h shifts and about 6 days per week, which is quite the norm for the majority of nurses in South Africa. These results suggest that the nurses who participated in this study fit the demographic profile of nurses in South Africa because there were no unusual features and patterns from the sample’s demographic characteristics.

This study used a three-point scale to measure burnout.^22^ Burnout level can either be low, moderate, or high. Findings indicate that just over half of the respondents (54%) had a high burnout level based on the EE measures. This finding was further substantiated by the results from the measures of DP, which showed that 78% of the respondents possibly experienced high-level burnout. High levels of burnout were also quite evident in the measures of reduced PA. These measures indicated that 72% of the respondents were likely experiencing high-level burnout.

The findings in this study supports recent studies that pointed to high levels of burnout among healthcare workers in South Africa. For example, one study recently highlighted the problem of burnout in the healthcare sector and found ‘that 40% of South African healthcare workers across the spectrum have suffered from burnout’.^23^

A systematic review of burnout among healthcare workers in sub-Saharan Africa also found that burnout is an issue that is currently receiving growing attention in research across the region.^10^ Burnout is experienced by all categories of healthcare workers, including doctors, nurses, midwives, and health professional students alike, as reviewed by Dubale et al.^10^

As the data for this study were collected just after the coronavirus disease 2019 (COVID-19) global pandemic, involving healthcare workers who were at the forefront of initiatives to curb the spread of the COVID-19 virus, higher than normal levels of burnout were to be expected. This could explain, to some degree, the high prevalence of high-level burnout among the respondents of this study.

However, it is also important to point out that burnout has been a huge concern in the healthcare industry long before the COVID-19 pandemic. For example, several studies going back to about a decade ago argue this issue that has been experienced in South Africa for much longer and COVID-19 simply drew attention to the issue.^23^ Another study conducted in the Western Cape province involving 42 doctors, which was published just before the COVID-19 pandemic, found that high levels of burnout on all three constructs (emotional exhaustion, DP, and reduced personal accomplishment)^15^ were present in 31% of the respondents.^24^

The studies cited above confirm the findings of this study, although burnout levels were quite high in this study’s sample.

Finally, this study also found certain characteristics, attitudes and symptoms that may be attributed to depression. About 18% of the respondents showed characteristics of severe depression. Furthermore, this study found that all the respondents may have experienced depression to varying degrees as a result of their work as nurses.

Findings from this study also indicate that there is a statistically significant positive correlation between burnout and depression. More specifically, these findings indicate that EE and DP (measures of burnout) increased simultaneously with depression in this sample.

Previous research has similarly found a strong relation between burnout and depression among healthcare professionals, which in turn was linked to substance abuse and relationship problems.^25^ In a similar manner, the study by Rossouw et al. showed burnout and depression among 132 medical doctors in Cape Town. The study found that from the 76% of the respondents that experienced burnout, 27% had signs and symptoms that indicated moderate depression, while 3% had severe depression.^26^ Much like this study, these studies establish a strong link between burnout and depression.

The multiple regression analysis conducted in this study revealed that burnout had an impact on depression. More specifically, the findings indicate that feelings associated with burnout such as DP, and years of working experience, had a notable impact on depression. As a result of the emotionally demanding nature of the nursing occupation, previous research has suggested that ‘nurses are twice as likely to suffer from depression than professionals in other fields’.^27^

Findings on the impact of burnout on depression (Mbanga et al.) similar to those of this study have been obtained in a cross-sectional study investigating whether burnout is a predictor of depression.^28^ Also some determinants of depression in nurses include the presence of burnout syndrome and longer durations of employment in the nursing profession.^28^

However, much of the available evidence in the literature is inconclusive about the nature of the relationship between burnout and depression. In fact, some of the scholars believe there is an overlap between the symptoms of burnout and depression.^7^ For example, Koutsimani et al. cited several studies that observed that people who suffer from burnout look and act as if they are depressed^7^ and noted that some of the burnout symptoms that appear similar to depression symptoms include loss of interest or pleasure, depressed mood, loss of energy, reduced concentration, and feelings of worthlessness, sleep problems and suicidal ideation.^28^ Mbanga et al. concur with these observations, highlighting that these overlapping symptoms include extreme exhaustion, depressed moods and reduced performance.^28^ These sources acknowledge that this overlap contributes to the usual difficulty in differentiating the two constructs and analysing the pathways of influence. These considerations are important as they might help explain why in this study only 52% of the variability in depression (dependent variable) has been significantly explained by burnout (independent variable).

## Conclusion

This study revealed a high prevalence of burnout measured as a second order construct through EE, DP, and reduced PA among nurses working at a Johannesburg private hospital. We also found a correlation between depression and EE, and DP. The study also revealed that DP and working experience had a statistically significant impact on depression. These findings extend our knowledge about the relationship between burnout and depression. The major contribution of this study is to raise awareness about burnout and depression. More research, preferably extensive longitudinal and prospective studies, is recommended to further explore the impact of burnout on depression and anxiety.

### Limitations of the study

There are some important limitations that need to be acknowledged in this study. Firstly, data were collected from only one hospital. Secondly, a small sample of 112 respondents participated in the study. Thirdly, there is a lack of representation of public hospitals in the study because the hospital involved in the study is a private hospital. Owing to this limitation, the findings presented in this study need to be interpreted cautiously because there are notable differences in working conditions between private and public hospitals.^29^ Also, because of the small sample, these findings cannot be generalised to other hospitals and populations beyond the delimitations of this study.
